# Analysis of SNP-SNP interactions and bone quantitative ultrasound parameter in early adulthood

**DOI:** 10.1186/s12881-017-0468-6

**Published:** 2017-10-03

**Authors:** María Correa-Rodríguez, Sebastien Viatte, Jonathan Massey, Jacqueline Schmidt-RioValle, Blanca Rueda-Medina, Gisela Orozco

**Affiliations:** 1 Faculty of Health Sciences, University of Granada, Av. Ilustración, 60, 18016 Granada, Spain; 2 Arthritis Research UK Centre for Genetics and Genomics, Centre for Musculoskeletal Research, Faculty of Biology, Medicine and Health, Manchester Academic Health Science Centre, The University of Manchester, Oxford Road, Manchester, M13 9PT UK

**Keywords:** Gene interaction, Quantitative ultrasound, Candidate gene

## Abstract

**Background:**

Osteoporosis individual susceptibility is determined by the interaction of multiple genetic variants and environmental factors. The aim of this study was to conduct SNP-SNP interaction analyses in candidate genes influencing heel quantitative ultrasound (QUS) parameter in early adulthood to identify novel insights into the mechanism of disease.

**Methods:**

The study population included 575 healthy subjects (mean age 20.41; SD 2.36). To assess bone mass QUS was performed to determine Broadband ultrasound attenuation (BUA, dB/MHz). A total of 32 SNPs mapping to loci that have been characterized as genetic markers for QUS and/or BMD parameters were selected as genetic markers in this study. The association of all possible SNP pairs with QUS was assessed by linear regression and a SNP-SNP interaction was defined as a significant departure from additive effects.

**Results:**

The pairwise SNP-SNP analysis showed multiple interactions. The interaction comprising SNPs rs9340799 and rs3736228 that map in the *ESR1* and *LRP5* genes respectively, revealed the lowest *p* value after adjusting for confounding factors (*p*-value = 0.001, β (95% CI) = 14.289 (5.548, 23.029). In addition, our model reported others such as *TMEM135-WNT16 (p* = 0.007, β(95%CI) = 9.101 (2.498, 15.704)*, ESR1-DKK1* (*p* = 0.012, β(95%CI) = 13.641 (2.959, 24.322) or *OPG-LRP5* (p = 0.012, β(95%CI) = 8.724 (1.936, 15.512). However, none of the detected interactions remain significant considering the Bonferroni significance threshold for multiple testing (p<0.0001).

**Conclusion:**

Our analysis of SNP-SNP interaction in candidate genes of QUS in Caucasian young adults reveal several interactions, especially between *ESR1* and *LRP5* genes, that did not reach statistical significance. Although our results do not support a relevant genetic contribution of SNP-SNP epistatic interactions to QUS in young adults, further studies in larger independent populations would be necessary to support these preliminary findings.

## Background

Osteoporosis represents an important public health problem worldwide that triggers near 9 million fractures annually [1]. Its prevalence is increasing due to the ageing of the population [2]. Osteoporosis is characterized by a deterioration of bone tissue microarchitecture that leads to low bone mineral density (BMD) and an increased risk of bone fragility fractures [3]. As occurs in many complex diseases, it is widely accepted that osteoporosis individual susceptibility is determined by the interaction of multiple genetic variants and environmental factors [4, 5].

An important determinant of osteoporosis risk later in life is the peak bone mass (PBM) [6]. Enhancing bone mass accrual to maximize PBM, which is attained by early adulthood, could help in the elderly to reduce the risk of fracture [6]. Current evidence suggests that genetic factors are major contributors to regulation of PBM, accounting for 50% and 80% of the variance in BMD [7, 8]. Therefore, identifying genetic factors affecting bone mass in early adulthood would be worthwhile.

Recently, it has been demonstrated that QUS predicts fracture independently of BMD [9, 10]. QUS provides information about bone mass and other quality aspects which may be of relevance in determining fracture risk such as bone microstructure, elasticity or connectivity [11, 12]. Besides this, previous studies have shown the ability of heel QUS to predict independently fracture risk [11, 13]. Compared to dual-energy x-ray absorptiometry (DXA), QUS is more easily accessible, low-cost, non-invasive and non-ionizing [14].

To identify the genetic factors underlying osteoporosis susceptibility, extensive genetic studies have been conducted reporting association of several genetic variants with different bone phenotypes [15–18]. However, these explain little of the heritability in complex phenotypes [19]. The missing heritability problem refers to the observation that the number of significant associations discovered does not form a substantial proportion of heritability for most traits. Thus, SNP-SNP interactions could help explain the missing heritability of common complex traits and therefore, their identification is considered of relevance. To date, only a few studies have been performed to identify SNP-SNP interactions influencing osteoporosis-related traits [20–26]. Most of them have investigated BMD determined by DXA, and therefore the possible influence of genetic interactions on QUS parameters is still unknown [25–27].

The independent association between single nucleotide polymorphisms (SNPs) in *WNT16* (rs2908007, rs2908004, and rs2707466) [18, 28, 29], *RSPO3* (rs774121) [18] and *LRP5* (rs3736228) [30] genes and QUS parameters have been well established. In previous replication studies from our group we investigated the implication of 32 genetic markers reported to influence QUS and/or BMD parameters, and confirmed that *WTN16*, *RSPO3* and *LRP5* are genetic factors that determine bone mineralization in young adults [28, 30].

Taking into consideration all this evidence, and bearing in mind that it is likely that interactions of common variants at different loci are influencing QUS traits, the aim of this study was to identify SNP-SNP interactions between SNPs independently associated with QUS, that could contribute to QUS traits variation in early adulthood.

## Methods

### Study subjects

A total of 575 Caucasian healthy individuals (400 females and 175 males, median age 20,41 ± 2,69 recruited from different centres of Granada (Spain) composed the study population after giving written informed consent. Local ethics committees approved the study that was conducted following the Declaration of Helsinki. Were excluded from the study those individuals who reported therapy with hormonal contraceptives, diseases of metabolic or endocrine systems and history of bone disease.

### Covariates

A body composition analyzer (TANITA BC-418MA) was used to estimate weight, fat mass and lean mass to the nearest 0,11 Kg. Height measurement was performed with a Harpenden stadiometer (Holtain 602VR®) to the nearest 0,1 cm. Body mass index (BMI) was calculated as weight divided by height squared (kg/m^2^). Physical activity (PA) was quantified using the short form and self-administered version of the International Physical Activity Questionnaire (IPAQ) [31]. Dietary calcium intake (DCI) was obtained from a 72 h recall method including intakes on Thursday, Friday, and Saturday. During the interviews and for a better precision of the food records, pictorial food models and standard household measures were used. All the 72 h recall were computerized with a nutrient analysis program (Nutriber 1.1.5) to convert food records into amounts of nutrients intake.

### QUS of the heel

Ultrasound measurements (BUA, dB/MHz) were performed using the CUBA clinical ultrasound bone densitometer (McCue Ultrasonic Limited, Compton, Winchester, UK) at the right calcaneus to assess bone mass. This localization was selected on the basis of its accessibility and its high content of trabecular bone [32]. To guarantee the long-term stability of the ultrasound bone densitometer, daily calibrations were performed using a with physical phantom.

### SNPs selection and genotyping process

A total of 32 SNPs mapping to loci that have been characterized as genetic markers for QUS and/or BMD parameters were selected.

DNA was extracted from saliva samples collected from all study participants with the 500 Collection Kit (DNA Genotek Inc., Ontario, Canada).

Genotyping was carried out using a custom design TaqMan OpenArray (Life Technologies, Carlsbad, CA, USA). Genotyping plate containing a predesigned TaqMan genotyping assay for every polymorphism was conducted in the Genomic and Genotyping unit of GENYO centre (Pfizer-University of Granada-Junta de Andalucía Centre for Genomics and Oncological Research). The amplification reaction was performed following the conditions recommended by the manufacturer. For detection of the fluorescence and interpretation of the genotyping results the QuantStudio 12 K Flex Real-Time PCR System (Applied Biosystems) was used.

All the Taqman assays included in the array showed a genotyping call rate above 95%. The accuracy of the genotyping process was verified including in all plates negative controls and duplicate samples. We observed 100% reproducibility.

### Statistical analysis

For all analyses the genotype of each SNP was encoded as 0, 1, 2, where 0 and 2 denotes homozygotes with major allele and minor allele respectively and 1 codes heterozygotes.

The Pearson’s goodness-of-fit χ^2^ test was used to assess the Hardy-Weinberg equilibrium (HWE). First, to reduce redundancy between SNPs, only one SNP out of all SNPs in strong pairwise linkage disequilibrium (r^2^ > 0.90) was kept, leading to the final inclusion of 30 SNPs (rs2707466 and rs7988338 were excluded) (Table 1). All possible pairwise SNP-SNP interactions between the selected 30 SNPs (435 possible interactions) were assessed by bivariate linear regression modelling of QUS measurements. Linear regression analyses were adjusted for the following covariates: sex, age, BMI, physical activity and calcium intake. The interaction between SNP_1_ and SNP_2_ was defined as the additional effect of their concomitant carriage on QUS over the addition of their independent effects (departure from additivity): β_interaction_ = β_observed_ – β_expected_ with β_expected_ = β_SNP1_ + β_SNP2_, where β_observed_ is the observed effect of the concomitant carriage of SNP_1_ and SNP_2_ (versus no carriage) in a bivariate linear regression, β_SNP1_ is the effect (β coefficient) of SNP_1_ in the absence of SNP_2_, β_SNP2_ is the effect of SNP_2_ in the absence of SNP_1_ in the same bivariate regression framework (bivariate refers only to the number of genetic markers (SNPs), as non-genetic covariates have systematically been included for all analyses). The results were reported as β_interaction_ (change of the outcome variable QUS with 95% confidence intervals (CIs). To all associations, the highly conservative Bonferroni correction considering the number of tested SNPs was applied. The cut-off value for significance was set at p<0.0001 (0.05/435). The STATA software was used to perform statistical analyses (version 11.0; STATA Corporation, College Station, TX).

**Table 1:** General information for the studied single nucleotide polymorphisms (SNPs)

Chromosome	Gene	SNP	Allele	MAF in this study	HWE (p)
2	*SPTBN1*	rs11898505	G > A	0.37	0.75
6	*RSPO3*	rs7741021	A > C	0.39	0.84
6	*CCDC170*	rs4869739	A > T	0.35	0.22
6	*ESR1*	rs3020331	C > T	0.43	0.10
6	*ESR1*	rs2982552	C > T	0.49	0.19
6	*ESR1*	rs2234693	T > C	0.44	0.19
6	*ESR1*	rs9340799	A > G	0.34	0.75
7	*WNT16*	rs2908007	T > C	0.18	0.33
7	*WNT16*	rs2908004	T > C	0.22	0.25
7	*WNT16*	rs3801387	T > C	0.10	0.78
7	*WNT16*	rs3801385	A > G	0.09	0.33
7	*WNT16*	rs2707466	G > A	0.22	0.39
7	*WNT16*	rs2536184	G > A	0.03	0.09
8	*OPG*	rs4355801	A > G	0.39	0.17
8	*OPG*	rs3102735	T > C	0.12	0.35
8	*OPG*	rs2073618	G > C	0.46	0.63
10	*DKK1*	rs7902708	G > C	0.11	0.77
11	*TMEM135*	rs597319	A > G	0.31	0.19
11	*LRP5*	rs2306862	C > T	0.19	0.32
11	*LRP5*	rs556442	A > G	0.42	0.88
11	*LRP5*	rs3736228	C > T	0.17	0.46
13	*RANKL*	rs9594759	T > C	0.48	0.40
13	*RANKL*	rs12585014	G > A	0.18	0.11
13	*RANKL*	rs7988338	G > A	0.19	0.40
13	*RANKL*	rs2148073	C > G	0.18	0.60
17	*SOST*	rs4792909	G > T	0.42	0.31
17	*SOST*	rs851054	A > G	0.38	0.06
17	*SOST*	rs2023794	T > C	0.05	0.18
18	*RANK*	rs1805034	C > T	0.41	0.26
18	*RANK*	rs12458117	G > A	0.19	0.35
18	*RANK*	rs3018362	A > G	0.32	0.18
19	*GPATCH1*	rs10416265	A > G	0.32	0.06

*MAF* minor allele frequency, *HWD* p value for Hardy-Weinberg equilibrium

## Results

The characteristics of the 575 study subjects have been published previously [28]. Table 1 shows marker information, including rsID and minor allele frequency (MAF) for 32 SNPs genotyped. All the SNPs were in HWE and none failed the frequency (MAF < 0.01) or missingess (genotyping >0.05) tests.

The pairwise SNP-SNP interactions tested by linear regression showed multiple interactions (Table 2). The interaction comprising SNPs rs9340799 and rs3736228 that map in the *ESR1* and *LRP5* genes respectively, revealed the lowest *p* value (0.001) after adjusting for sex, age, BMI, physical activity and calcium intake. This interaction amounted to a β (95% CI) = 14.289 (5.548, 23.029). Interestingly, we found several interactions between different polymorphisms in these genes, further supporting the presence of epistasis between these two loci (rs556442 in *LRP5* and rs9340799 in *ESR1;* rs2234693 in *ESR1* and rs3736228 in *LRP5;* rs2306862 in *LRP5* and rs9340799 in *ESR1*; rs556442 in *LRP5* and rs2234693 in *ESR1*). However, none of the pairwise SNP-SNP tests reached the significant cut off *p* value considering the Bonferroni correction for multiple testing (*p* = 0.0001).

**Table 2:** Gene-gene interaction analysis

SNP1	Gene 1	SNP2	Gene 2	β (95% CI)	*p*-value
rs9340799	*ESR1*	rs3736228	*LRP5*	14.289 (5.548, 23.029)	0.001
rs2908004	*WNT16*	rs3801387	*WNT16*	77.350 (25.890, 128.808)	0.003
rs597319	*TMEM135*	rs2908004	*WNT16*	9.101 (2.498, 15.704)	0.007
rs3736228	*LRP5*	rs2306862	*LRP5*	23.476 (5.623, 41.327)	0.010
rs9340799	*ESR1*	rs7902708	*DKK1*	13.641 (2.959, 24.322)	0.012
rs4355801	*OPG*	rs556442	*LRP5*	8.724 (1.936, 15.512)	0.012
rs3801387	*WNT16*	rs2982552	*ESR1*	−8.705 (−15.554, −1.855)	0.013
rs556442	*LRP5*	rs9340799	*ESR1*	9.859 (2.045, 17.673)	0.013
rs11898505	*SPTBN1*	rs10416265	*GPATCH1*	−9.200 (−16.751, −1.649)	0.017
rs2982552	*ESR1*	rs3020331	*ESR1*	13.312 (2.242, 24.381)	0.018
rs2148073	*RANKL*	rs1805034	*RANK*	8.443 (1.127, 15.758)	0.023
rs4869739	*CCDC170*	rs7741021	*RSPO3*	−7.748 (−14.501, −0.995)	0.024
rs2234693	*ESR1*	rs3736228	*LRP5*	10.606 (1.361, 19.851)	0.024
rs556442	*LRP5*	rs12458117	*RANK*	−9.760 (−18.261, −1.258)	0.024
rs3801387	*WNT16*	rs4355801	*OPG*	7.333 (0.887, 13.777)	0.025
rs2306862	*LRP5*	rs9340799	*ESR1*	8.239 (0.973, 15.504)	0.026
rs2982552	*ESR1*	rs9340799	*ESR1*	9.322 (1.037, 17.606)	0.027
rs7902708	*DKK1*	rs4355801	*OPG*	−8.159 (−15.383, −0.934)	0.027
rs2306862	*LRP5*	rs4355801	*OPG*	7.779 (0.888, 14.668)	0.027
rs2306862	*LRP5*	rs11898505	*SPTBN1*	7.989 (0.793, 15.183)	0.029
rs556442	*LRP5*	rs2234693	*ESR1*	7.449 (0.664, 14.233)	0.031
rs3801385	*WNT16*	rs2982552	*ESR1*	−11.658 (−22.351, −0.965)	0.032
rs9340799	*ESR1*	rs7741021	*RSPO3*	7.191 (0.409, 13.973)	0.037
rs597319	*TMEM135*	rs851054	*SOST*	6.828 (0.405, 13.25)	0.037
rs2148073	*RANKL*	rs10416265	*GPATCH1*	−7.879 (−15.478, −0.278)	0.042
rs597319	*TMEM135*	rs2908007	*WNT16*	6.889 (0.197, 13.579)	0.043
rs3801387	*WNT16*	rs12458117	*RANK*	7.949 (0.192, 15.704)	0.044
rs2234693	*ESR1*	rs3801385	*WNT16*	13.418 (0.273, 26.562)	0.045
rs3018362	*RANK*	rs10416265	*GPATCH1*	7.359 (0.114, 14.604)	0.046
rs12458117	*RANK*	rs7741021	*RSPO3*	−7.388 (−14.744, −0.031)	0.048
rs2908004	*WNT16*	rs4355801	*OPG*	7.273 (0.014, 14.530)	0.049

SNP1 and SNP2 indicate the individual SNPs within a given SNP-SNP interaction model. Beta represents the regression coefficient. *P* values are shown adjusted for the covariates sex, age, BMI, physical activity and calcium intake

On the other hand, our analyses revealed SNP-SNP interactions within the same genes (rs2908004 and rs3801387 in *WNT16;* rs3736228 and rs2306862 in *LRP5;* rs2982552 and rs3020331 in *ESR1*; rs2982552 and rs9340799 in *ESR1*).

## Discussion

In this study, we aimed to provide a more comprehensive view of the genetic architecture underlying QUS by analysing two-way interactions between common SNPs in candidate genes. A number of SNPs in candidate genes were analysed considering potentially important covariates for bone mineralization process such as, age, sex, calcium intake, physical activity and BMI.

This study is the first to investigate SNP-SNP interactions with heel QUS trait. Our findings identified several SNP-SNP interactions, highlighting that observed between rs9340799 in *ESR1* and rs3736228 in *LRP5.* However, the results from these analyses should be interpreted with caution since SNP-SNP interactions did not reach the highly conservative Bonferroni significance threshold for multiple testing (p<0.0001). Even though these data suggest that epistatic SNP-SNP interactions does not influence heel ultrasound measurements in young Caucasian adults, the possibility of a false negative due to a limited statistical power cannot be excluded.

Oestrogen action in bone are mediated mainly through ERα codified by the *ESR1* gene, which has been associated with different osteoporosis related traits in previous studies [33]. The positive effects of the oestrogens on the skeleton have been well established; oestrogens play a major role in the aetiology of osteoporosis by the regulation of bone turnover and inhibition of bone loss [34]. These biological effects are mediated by binding and activation of specific oestrogen receptors (ERα and ERβ) [35]. The rs9340799 variant is located in the first intron of the *ESR1* gene and although this intron may contain regulatory elements, the functional implication of this genetic variant remains unknown. In addition, low-density lipoprotein receptor-related protein 5 (*LRP5*) codifies a protein belonging to the Wnt canonical signal, which regulates osteoblast and osteocyte function [36]. Similarly, the functional relevance of the rs3736228 SNP located in *LRP5* is still unknown [37, 38].

The results of this study could be suggestive of a well-regulated cross-talk between *ESR1* and *LRP5* genes in bone physiology. Although *ESR1* and *LRP5* develop different roles in skeletal homeostasis, they could be interacting in bone cell biology through synergistic or antagonistic actions. It could be hypothesised that individuals carrying risk alleles at rs9340799 and rs3736228 SNPs have a lower expression of both *ESR1* and *LRP5,* which could lead to an impaired bone mass. As we have analysed a cohort including only young adults, our findings also may suggest that the interaction between rs9340799 and rs3736228 SNPs might be implicated in bone mass accrual. Although previous studies have reported the individual contribution of these polymorphisms to osteoporosis-related phenotypes in early adulthood [30, 38–41], the molecular mechanisms through which these polymorphisms might interact are still under investigation. Future studies are required to elucidate the molecular mechanisms by this interaction is implicated in the bone mass acquisition process during early adulthood.

Besides the interaction between *LRP5* and *ESR1*, our model suggested others such as *TMEM135*-*WNT16*, *ESR1-DKK1* or *OPG-LRP5*. Interestingly, all of these genes belong to well characterize pathways implicated in the complex mechanisms that regulate bone formation. Thus, a novel line of research would be very interesting to elucidate how they may be interacting at the molecular level.

On the other hand, SNP-SNP interactions between variants that were not in LD within the same gene were observed. That was the case of genes *WTN16, LPR5* and *ESR1*. Thus, beyond the individual effect of SNPs in a gene, epistatic SNP-SNP interactions in the same gene occur and could be a potential factor contributing to the unexplained heritability of bone mass acquisition.

On the other hand, it could be hypothesised that the effects of SNP-SNP interactions would be weak and therefore, a very large population size would be needed to detect associations surpassing the *p* value threshold for multiple testing (p<0.0001). Thus, a limitation of the present study could be the population size included that would not reach enough statistical power to detect epistatic SNP-SNP interactions with weak effect. Therefore, in order to support our preliminary results, further studies including larger population size and meta-analysis are needed. On the other hand, the current approach considered only two-locus interactions and therefore more complex interactions between three or more genetic markers are unknown. Again, larger studies are required to confirm our findings and to elucidate mechanisms by which the genetic interaction between these genes influences quantitative bone phenotypes in early adulthood.

## Conclusions

Our analysis of SNP-SNP interaction in candidate genes of QUS in Caucasian young adults reveal several interactions, especially between *ESR1* and *LRP5* genes, that did not reach statistical significance. Although our results do not support a relevant genetic contribution of SNP-SNP epistatic interactions to QUS in young adults, further studies in larger independent populations would be necessary to support these preliminary findings.

## Abbreviations

BMD: Bone mineral density
BMI: Body mass index
BUA: Broadband ultrasound attenuation
CIs: Confidence intervals
DCI: Dietary calcium intake
DXA: Dual-energy x-ray absorptiometry
GWAS: Genome wide association studies
HWE: Hardy-Weinberg equilibrium
IPAQ: International Physical Activity Questionnaire
MAF: Minor allele frequency
PBM: Peak bone mass
QUS: quantitative ultrasound
